# How to improve medical education website design

**DOI:** 10.1186/1472-6920-10-30

**Published:** 2010-04-21

**Authors:** Stephen D Sisson, Felicia Hill-Briggs, David Levine

**Affiliations:** 1 Department of Medicine, Johns Hopkins University School of Medicine, 600 N. Wolfe Street, Baltimore 21287, USA

## Abstract

**Background:**

The Internet provides a means of disseminating medical education curricula, allowing institutions to share educational resources. Much of what is published online is poorly planned, does not meet learners' needs, or is out of date.

**Discussion:**

Applying principles of curriculum development, adult learning theory and educational website design may result in improved online educational resources. Key steps in developing and implementing an education website include: 1) Follow established principles of curriculum development; 2) Perform a needs assessment and repeat the needs assessment regularly after curriculum implementation; 3) Include in the needs assessment targeted learners, educators, institutions, and society; 4) Use principles of adult learning and behavioral theory when developing content and website function; 5) Design the website and curriculum to demonstrate educational effectiveness at an individual and programmatic level; 6) Include a mechanism for sustaining website operations and updating content over a long period of time.

**Summary:**

Interactive, online education programs are effective for medical training, but require planning, implementation, and maintenance that follow established principles of curriculum development, adult learning, and behavioral theory.

## Background

A curriculum is a planned learning experience, and the Internet has grown as a resource for disseminating curricula, including medical education curricula [1,2]. Advantages of Internet-based curricula include flexibility in training times, adaptability to learner styles, interactivity, access by geographically dispersed learners, communication between learners and educators, and hyperlinks [1,3]. When developed properly, internet-based medical education courses can result in knowledge gains similar to, or even superior to, more traditional teaching methods [3,4]. In a meta-analysis of studies of online learning, the United States Department of Education found that student performance was better with online instruction when compared to face-to-face instruction [5]. Unfortunately, many curricula published online do not follow principles of curriculum development, established learning principles, or use a systematic approach to education [6-13]. Much of what is published online is poorly planned, difficult to adapt to unanticipated needs, and is out of date [14].

**Figure 1:**
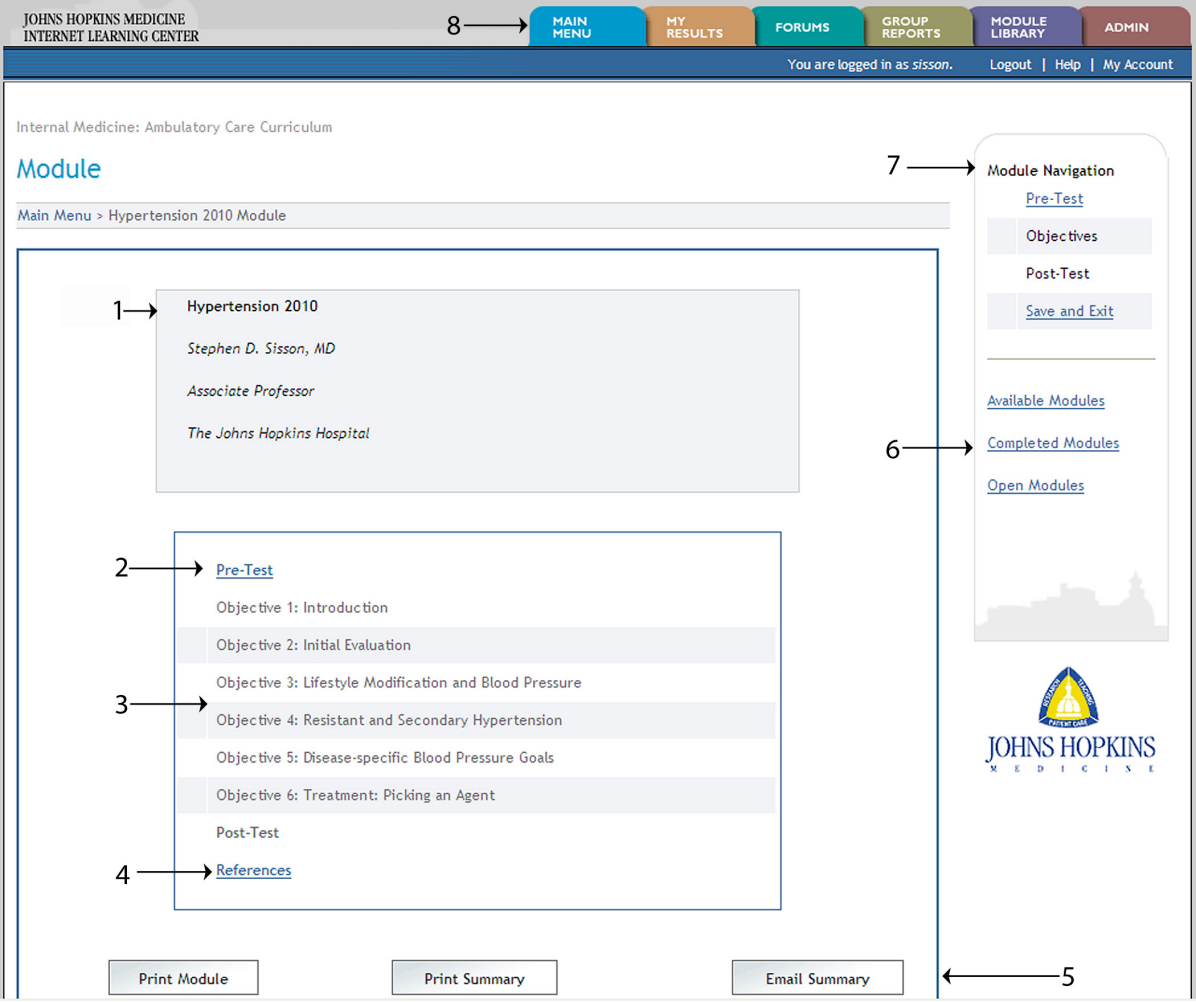
**Module navigation page**. Learner selects module from list of topics on welcome page, which directs to module navigation page. Module topic is displayed on module navigation page (1). Modules are stuctured with both program control and learner control. To start a module, the learner must complete the pretest (2), which then activates the didactics section (3). The learner may complete didactics in the order of his/her choosing. The post test is then activated once all objectives of didactics are complete. Links to references may be accessed at any time (4). Completion of the entire module enables the learner to print the didactics section or a module summary, or have the summary emailed to their address (5). Website navigation includes access to other modules (6) or the current module (7). Tabs at the top of the page (8) access the main menu or individual test results. Learners can also post messages and communicate with other learners in a Forums section (i.e. "chat rooms"). Administrators at each training program have additional tabs (8) to access trainee and performance results, and to schedule modules according to their needs. The webmaster and curriculum editors have an additional administrative tab to approve/delete registrants and training programs, as well as upload educational content.

**Figure 2:**
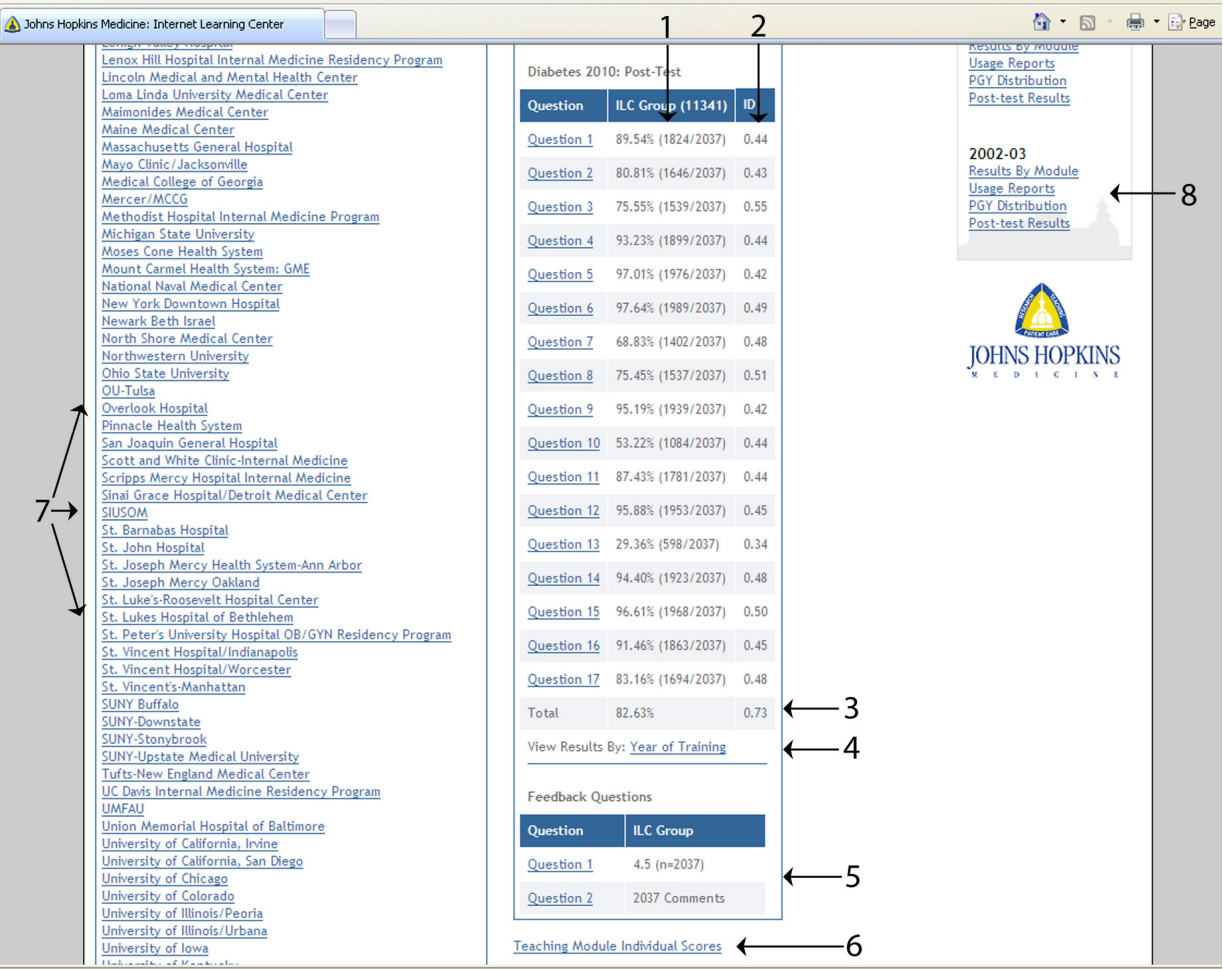
**Main Results page**. Pretest and post test performance is calculated as modules are completed and can be accessed by administrators at each participating program. Performance on each item is calculated (1) along with item discrimination (2). Results may be drilled down to include frequency of incorrect answers on each item. Cronbach's alpha is calculated on each pretest and post test (3). Results can also be viewed by year of training (4). A feedback section displays learner ratings on the instructive value of the module, and individual comments on how to improve the module can be accessed (5). For statistical calculations, individual scores grouped by year of training can be accessed (6). Performance may be drilled down for each training program (7), although each training program can view only their scores and aggregate scores of all participating programs. Performance on prior years can also be accessed (8).

At its best, online learning should be "flexible, engaging, and learner-centered"[15]. Since such high quality online educational resources are expensive to produce and maintain, well designed educational programs that are shared across multiple institutions allow the sharing of expenses, while meeting diverse learner and institutional needs and generating benchmarks of competency in medical education and evaluation [12,16]. The Johns Hopkins Internet Learning Center is a website that has hosted a curriculum in ambulatory care since 2001. The curriculum is composed of topic-specific modules (e.g., Cancer Screening, Immunizations, Hypertension, and Back Pain) that are divided into a pretest, case-based didactics, and a post test (Figure 1). Residency training programs subscribe to this curriculum by paying an annual fee, enabling their residents to register and complete the training modules. Training modules are primarily text-based, supported with images, figures, videos, and links to medical references. Each module is interactive, provides immediate feedback, but requires the learner to complete the pretest before the didactics, and to complete the didactics before the post test. Learners and program directors can track their performance and compare their performance relative to other trainees and programs (Figure 2). Learners are required to provide feedback on the instructive value of the module and how to improve it. The website calculates performance by learning objective, and does reliability and validity testing on the pretest and post test with item discrimination (which assesses reliability of test questions), Cronbach's alpha (which assesses reliability of an entire test), and year-of-training performance (which provides construct validity to test results). Feedback from learners and performance outcomes are reviewed annually as a basis for module revision. Fees paid by subscribing programs are used to pay for website support and educational content.

When first launched in 2001, 24 residency training programs subscribed to the curriculum. Since then, that number has grown to 133 programs, including 8332 resident and 1867 attending physicians. We review here the education theories and website design principles that we found most important in developing our website, to provide a guide on how to improve medical education website design.

## Discussion

### The role of education theory and website design

For a website to serve as a successful educational resource, developers should combine established principles of curriculum development, adult learning and behavioral theory, along with principles of website design [12].

#### Curriculum development

The educational content of a well-planned curriculum has more impact on learner satisfaction than the technology used to disseminate it [4]. The Internet provides a mechanism of rapid dissemination of a potentially poorly designed curriculum; merely publishing it online does not make a curriculum good [7,11]. Curricula produced using established principles of curriculum development are more likely to be successfully implemented than those that are not [2,10]. The six steps of curriculum development are as follows [2]:

1. Identify a problem and perform a general needs assessment

2. Perform a needs assessment of targeted learners

3. Determine goals and objectives of curriculum

4. Determine educational strategies to be used with curriculum

5. Implement the curriculum

6. Evaluate the curriculum

Performing a needs assessment is the first step of curriculum development, and is essential to creating a successful education website [2]. There are several potential targets of a needs assessment including learners, educators, administrators, and society [2]. Those curricula that serve the needs of multiple audiences are more likely to succeed than those serving the needs of a narrow segment [2]. A curriculum that is too specialized or too narrowly focused may fail to find an audience large enough to justify the expense of development. Our initial needs assessment included national surveys of ambulatory care, which we used to choose topics for content development [16]. As a result, our curriculum met the needs of a large number of training programs, and contributed to the success of our curriculum [17].

The needs assessment should not only be done during the beginning stages of curriculum development, but incorporated as a recurring process as the curriculum is revised. The needs of the target audiences will evolve over time; a mechanism to regularly gather this information for use to update the curriculum should be included in curriculum maintenance [7,18]. Repeated needs assessments are likely to reveal unanticipated needs, and if acted upon will improve the curriculum [19]. In a sense, curriculum evaluation, defined as the final step of curriculum development, can include an evaluation of the needs of current users, and serve as a needs assessment for the next iteration of the curriculum.

Our website provides users (learners and program directors) several opportunities to comment on how the curriculum meets their needs. Each module requires that learners rate the instructive value of the module, and closes with a free-text feedback box prompting learners to comment on how to improve the module (Figure 2). Learners and program directors can also post comments on the curriculum and website in "chat rooms" provided on the website, or email comments or complaints directly to the webmaster or curriculum developers through a "contact us" feature. We have found that most learners prefer to comment in the feedback section, and few use the chat rooms or contact us directly. Many modules have undergone significant revision based on these comments, with a resultant increase in these modules' ratings on instructive value.

#### Adult learning theory and content development

Principles of adult learning theory echo the importance of the needs assessment in educational websites, and we used the theories of Malcolm Knowles to guide content development, listed below [6,20,21].

1. Adult learners are autonomous and self directing

2. Adult learners bring with them a great range of prior experience

3. The necessity to learn something must be clear to the adult learner before they will commit to learning it

4. Readiness to learn in the adult learner is greatly impacted by what the learner thinks they need to know to cope effectively with real life situations

5. Adult learners are motivated by problem-centered or task-centered education

6. The strongest motivators for the adult learner have intrinsic value rather than extrinsic value

Adult learners are typically autonomous and self-directed, and are motivated by education that has intrinsic value to personal goals and a sense of self [21]. Curricular material should be presented in such a context that the adult learner will understand its importance and relevance to his or her own needs. Adult learners are also motivated by problem-centered or task-centered education, which should consist of realistic scenarios that they may anticipate encountering.

We applied these theories of adult learning when we chose our goals/objectives and educational strategies (i.e. steps 3 and 4 of curriculum development). We chose a case-based, problem-solving format for our curriculum, with patient scenarios similar to what we knew residents were seeing in clinic. Nearly all modules were written by clinically active general internists who precepted the residents in clinic, and as a result (based on comments in the feedback section), case scenarios used in the curriculum resonated with the residents as they completed the modules.

#### Website design principles

Aspects of educational website design rival content in impacting learner satisfaction, demonstrate the importance of proper implementation (step 5 of curriculum development), and should follow established guidelines of website design, as noted below [4,22].

1. Use words and graphics in a balanced and contiguous manner

2. Use conversational style to text

3. Consider use of audio, but not that which is redundant to text

4. Avoid extraneous audio, graphics, hyperlinks, and text

5. Use realistic job context to teach problem-solving

6. Learners prefer to control the order of content, but beginners and complex material may require fixed progression through content

Someone with skill in website design and the technical aspects of website function should be included in planning educational websites so that functionality will be assured regardless of internet browser or connectivity issues [4,12,16]. Well-designed websites can successfully introduce complex subjects and may reduce the time needed to learn a specific objective [7,23,24]. There is some evidence that well-designed computer-assisted instruction may be more effective than traditional forms of instruction; in one study, average post test scores were raised from the 50^th ^to the 60^th ^percentile using computer-assisted instruction, and another showed long-term knowledge gains similar or superior to knowledge gains from live lectures [3,4,24].

Medical education websites should take advantage of features specific to the Internet, integrating content with capabilities made possible online [11]. Content should be presented in a format that is interactive, combining words and graphics that are balanced so as to avoid visually overwhelming the learner [6,20,22]. Educational content that is presented as review material should allow learners to control the sequence of learning (i.e. "learner control"), while more complicated content should be introduced in a specific order (i.e. "program control") [6,22]. Complex educational websites incorporate "adaptive control", for which the content adjusts based on learner performance. Adaptive control is very resource-intensive to produce [22]. Basic navigation (e.g. forward/back, main menu, save/exit) should be included on every web page. The technology offered by the Internet should only be used in ways that enhance the educational experience [18].

In our experience, we receive many more comments on the formatting of content than we do on the content itself. Learners frequently ask for more tables, diagnostic algorithms, and images to support the text. Our website combines learner control and program control. Didactics cannot be accessed until the pretest is completed, but then the learner can progress through the didactics in an order of their choosing. The post test cannot be accessed until each section of the didactics has been completed. This combination maintains validity of the pretest/post test comparisons, but allows the learner autonomy in completing the didactics. Some learners object to the program control aspects of our curriculum, most frequently wishing to bypass the pretest, or to allow viewing of didactic content before completing the pretest. Learners requested a printable summary page, which was added three years ago. More recently, several learners have requested smart phone capabilities of the website, but has not yet been addressed due to cost.

Links to relevant external resources are an obvious attraction of medical education websites [11,12,23]. However, too many links can overwhelm the learner and are frequently bypassed [22]. In our experience, a minority access external links, but those who do comment that they are essential. Other potential features of educational websites include video and audio didactics. When designing an educational website, applications used should be those that are in widespread use [12]. Learners accessing content from medical schools, hospital computers or public workstations may not have the option to download new software or have sound or print capabilities. Our website was designed to be compatible with all internet browsers and allows users to email summary pages to themselves should they not have access to a printer when using the curriculum. We found excellent audiovisual resources on a popular video website http://www.youtube.com, and linked to those resources in some modules.

### Education outcome assessment

The Institute of Medicine has noted the link between the quality of healthcare and the quality of medical education [25]. The Society of Directors of Research in Medical Education and others have also identified demonstration of the effectiveness of medical education as a priority [7,26-28]. As medical schools and residency training programs strive to demonstrate competence among their trainees, educators and institutions will need the tools to obtain reliable and valid results. As independent development of education assessment tools is expensive, developing a medical education website that also measures educational effectiveness and demonstrates the reliability of the results enhances the value of the educational website. Linking of teaching and assessment is possible with educational websites, and should be exploited to fulfill the needs of institutions and accreditation bodies [7,26,29].

Educational websites should incorporate methods to allow for assessment of both the learner and the content. The most basic evaluation that can be provided by an interactive website is formative and summative feedback to the learner as they complete clinical problem solving exercises. Demonstrating the reliability and validity of these results, both on an individual learner and a programmatic level, requires more resources [29-33]. However, by developing validated outcomes measures, developers have the opportunity to set performance standards on knowledge and skills that may serve as a benchmark for judging trainee or physician performance [23,33]. Currently, benchmarking is in its early stages of development; proliferation of well-designed educational websites may assist with standard setting and create a range of validated outcome measurement tools for the medical educator [1,33].

We chose a pretest-intervention-post test structure so that educational effectiveness could be measured (the pretest and post test are measured for equivalence during development). Item discrimination and Cronbach's alpha are used for reliability testing (Figure 2). Residency programs can set a pass/fail score that their residents must obtain to demonstrate competency on the subject. Individual module performance is grouped by competency, and an individual learner's competency score is compared to aggregate scores of all learners to provide each individual a rank score for the competency. Each component of evaluation required significant resources to develop, but sharing of our curriculum provides participating training programs useful evaluation of their trainees automatically. The large number of training programs using our curriculum suggests that aggregate results may provide benchmarks of performance among trainees.

### Other issues

#### The importance of sustainability

Medical schools and residency training programs should consider an educational website's sustainability before incorporating it into their training [9]. In the early 1990s, dozens of medical schools had developed online educational resources, but by the end of the decade, most of them were no longer being maintained [19]. Many currently active educational websites are not being updated or improved [8]. Since successful programmatic use of an educational website requires that it be fully embedded and supported within a training experience, program leaders are unlikely to incorporate a product that is not current [7,8]. Therefore, when designing an educational website, plans should include how the website is to be maintained over time and how often content should be updated [11]. Planning for long-term resource commitment to the website, not only for day to day operations and future website enhancements, but also a mechanism for updating educational content, should be considered as any new website is developed [14,17].

We chose to hire a professional webmaster to oversee day to day operations of the website. Module authors are required to update content annually. Curriculum editors review feedback suggestions several times throughout the year, using these suggestions as a basis for annual website and curriculum upgrades. Financial support to sustain the curriculum is provided by charging residency programs an annual fee for access to the curriculum, which is used to pay the webmaster, support upgrades, and pay module authors and editors for content.

#### Academic recognition

Perhaps overlooked, yet vital to the quality of the educational content of the website, are the needs of the educators who write the actual content of the curriculum. To ensure outstanding curricular content, educators should be recognized for their efforts, typically in the form of promotion. However, many educators are unsure of how tenure and promotions committees recognize teaching materials developed for educational websites [34]. Department chairs and promotions committees can contribute to the success of an educational website by establishing criteria for recognition of authoring educational content that is disseminated online [35].

In our experience, lack of academic recognition for curriculum module authorship remains a barrier to recruiting faculty to write modules, and is partially overcome by the possibility of evaluating the educational effectiveness of a module and submitting it for publication. We also use funds paid by subscribing programs to pay authors for content, although academic recognition remains the batter motivator.

## Summary

In developing online educational resources, the following guidelines are suggested:

1. Principles of curriculum development should be used when developing educational resources, starting with a needs assessment.

2. Principles of adult learning should be considered as educational content, educational strategies, and website function are developed.

3. Websites should be designed to demonstrate the educational effectiveness of their intervention, both on an individual and a programmatic level.

4. Website development should include a mechanism for sustaining website operations, repeating the needs assessment, and updating content over a long period of time.

## Competing interests

The authors declare that they have no competing interests.

## Authors' contributions

SS contributed to the conception and design, as well as the analysis of the topics discussed herein. SS also drafted the original manuscript along with incorporated revisions suggested by other authors. SS has given approval to the final version of the manuscript. FH-B contributed to the conception of the manuscript and the analysis of the topics. FH-B critically reviewed the manuscript and gave approval to the final version of it. DL contributed to the conception of the manuscript and the analysis of the topics. DL critically reviewed the manuscript and gave approval to the final version of it.

## Pre-publication history

The pre-publication history for this paper can be accessed here:

http://www.biomedcentral.com/1472-6920/10/30/prepub

## References

[B1] Harden RM Hart IR An international virtual medical school (IVIMEDS): the future for medical education? Med Teacher 2002 24 261 7 10.1080/01421590220141008 12098412

[B2] Kern DE Thomas PA Howard DM Bass EB Curriculum development for medical education: A six-step approach 1998 Johns Hopkins University Press, Baltimore

[B3] Fordis M King JE Ballantyne CM Jones PH Schneider KH Spann SJ Greenberg SB Greisinger AJ Comparison of the instructional efficacy of Internet-based CME with live interactive CME workshops: A randomized controlled trial JAMA 2005 294 1043 51 10.1001/jama.294.9.1043 16145024

[B4] Chumley-Jones HS Dobbie A Alford CL Web-based learning: Sound educational method or hype? A review of the evaluation literature Acad Med 2002 77 S86 S93 10.1097/00001888-200210001-00028 12377715

[B5] US Department of Education, Office of Planning, Evaluation, and Policy Development Evaluation of evidence-based practices in online learning: A meta-analysis and review of online learning studies. Washington, DC 2009 United States Department of Education http://www2.ed.gov/rschstat/eval/tech/evidence-based-practices/finalreport.pdf

[B6] Harden RM A new vision for distance learning and continuing medical education J Cont Educ Health Prof 2005 25 43 51 10.1002/chp.8 16078802

[B7] Ward JPT Gordon J Field MJ Lehman HP Communication and information technology in medical education Lancet 2001 357 792 96 10.1016/S0140-6736(00)04173-8 11253986

[B8] Friedman RB Top ten reasons the World Wide Web may fail to change medical education Acad Med 1996 71 979 81 9125986 10.1097/00001888-199609000-00013

[B9] Green ML Identifying, appraising, and implementing medical education curricula: A guide for medical educators Ann Intern Med 2001 135 889 96 11712879 10.7326/0003-4819-135-10-200111200-00009

[B10] Windish DM Gozu A Bass EB Thomas PA Sisson SD Howard DM Kern DE A ten-month program in curriculum development for medical educators: 16 years of experience J Gen Intern Med 2007 22 655 61 10.1007/s11606-007-0103-x 17443374 PMC1852913

[B11] Cook DA Dupras DM A practical guide to developing effective Web-based learning J Gen Intern Med 2004 19 698 707 10.1111/j.1525-1497.2004.30029.x 15209610 PMC1492389

[B12] Greenhalgh T Computer assisted learning in undergraduate medical education BMJ 2001 322 40 4 10.1136/bmj.322.7277.40 11141156 PMC1119309

[B13] Alur P Fatima K Joseph R Medical teaching websites: do they reflect the learning paradigm? Med Teacher 2002 24 422 4 10.1080/01421590220145815 12193328

[B14] Candler CS Uijtdehaage SHJ Dennis SE Introducing HEAL: The Health Education Assets Library Acad Med 2003 78 249 53 10.1097/00001888-200303000-00002 12634201

[B15] Ellaway R Masters K AMEE Guide 32: e-learning in medical education part 1: Learning, teaching, and assessment Med Teach 2008 30 455 73 10.1080/01421590802108331 18576185

[B16] Sisson SD Hughes MT Levine D Brancati FL Effect of an Internet-based curriculum on postgraduate education: A multicenter intervention J Gen Intern Med 2004 19 505 9 10.1111/j.1525-1497.2004.30097.x 15109313 PMC1492333

[B17] Sisson SD Rastegar DA Rice TN Hughes MT Multicenter implementation of a shared graduate medical education resource Arch Intern Med 2007 167 2476 80 10.1001/archinte.167.22.2476 18071170

[B18] Whitcomb ME The information technology age is dawning for medical education Acad Med 2003 78 247 8 12634200 10.1097/00001888-200303000-00001

[B19] Salas AA Anderson B LaCourse L Allen R Candler CS Cameron T Lafferty D CurrMIT: A tool for managing medical school curricula Acad Med 2003 78 275 9 10.1097/00001888-200303000-00009 12634208

[B20] Davis D O'Brien MAT Freemantle N Wolf FM Mazmanian P Taylor-Vaisey A Impact of formal continuing medical education. Do conferences, workshops, rounds, and other traditional continuing education activities change physician behavior or health care outcomes? JAMA 1999 282 867 74 10.1001/jama.282.9.867 10478694

[B21] Knowles MS Holton EF Swanson RA The Adult Learner 1998 Fifth Butterworth-Heinemann. Woburn, MA

[B22] Clark RC Mayer RE e-Learning and the Science of Instruction 2003 J Wiley and Sons, Inc. San Francisco

[B23] Schultze-Mosgau S Zielinski T Lochner J Web-based, virtual course units as a didactic concept for medical teaching Med Teach 2004 26 336 42 10.1080/01421590410001679028 15203847

[B24] Baumlin KM Bessette MJ Lewis C Richardson LD EMCyberSchool: An evaluation of computer-assisted instruction on the Internet Acad Emer Med 2000 7 959 62 10.1111/j.1553-2712.2000.tb02083.x 10958144

[B25] Institute of Medicine Crossing the Quality Chasm: A new health system for the 21^st ^Century 2001 Washington, DC; National Academy Press

[B26] Report of the Ad Hoc Committee of Deans Educating doctors to provide high quality medical care: A vision for medical education in the United States American Association of Medical Colleges 2004 Association of American Medical Colleges http://services.aamc.org/publications/showfile.cfm?file=version132.pdf&prd_id=262&prv_id=321&pdf_id=132

[B27] Accreditation Council for Graduate Medical Education. ACGME Outcome Project http://www.acgme.org 30 October 2007

[B28] Cook DA The failure of e-learning research to inform educational practice, and what we can do about it Med Teach 2009 31 158 62 10.1080/01421590802691393 19330674

[B29] Wolf FM Shea JA Albanese MA Toward setting a research agenda for systematic reviews of evidence of the effects of medical education Teach Learn Med 2001 13 54 60 10.1207/S15328015TLM1301_11 11273381

[B30] Goroll AH Sirio C Duffy D LeBlond RF Alguire P Blackwell TA Rodak WE Nasca T A new model for accreditation of residency programs in internal medicine Ann Intern Med 2004 140 902 9 15172905 10.7326/0003-4819-140-11-200406010-00012

[B31] Holmboe ES Hawkins RE Methods for evaluating the clinical competence of residents in internal medicine: a review Ann Intern Med 1998 129 42 8 9652999 10.7326/0003-4819-129-1-199807010-00011

[B32] Weinberger SE Smith LG Collier VU Redesigning training for internal medicine Ann Intern Med 2006 144 927 32 16601254 10.7326/0003-4819-144-12-200606200-00124

[B33] Fitzgibbons JP Bordley DR Berkowitz LR Miller BW Henderson MC Redesigning residency education in internal medicine: a position paper from the Association of Program Directors in Internal Medicine Ann Intern Med 2006 144 920 6 16785480 10.7326/0003-4819-144-12-200606200-00010

[B34] Davis MH Harden RM Competency-based assessment: Making it a reality Med Teach 2003 25 565 68 10.1080/0142159032000153842 15369903

[B35] Uitdehaage SHJ Contini J Candler CS Dennis SE Sharing digital teaching resources: Breaking down barriers by addressing concerns of faculty members Acad Med 2003 78 286 94 12634210 10.1097/00001888-200303000-00011

